# Influence of Pedometer Position on Pedometer Accuracy at Various Walking Speeds: A Comparative Study

**DOI:** 10.2196/jmir.5916

**Published:** 2016-10-06

**Authors:** Frederic Ehrler, Chloé Weber, Christian Lovis

**Affiliations:** ^1^Division of Medical Information SciencesUniversity Hospitals of GenevaGenevaSwitzerland; ^2^Faculty of MedicineUniversity of GenevaGenevaSwitzerland

**Keywords:** frail elderly, mHealth, walking, motor activity

## Abstract

**Background:**

Demographic growth in conjunction with the rise of chronic diseases is increasing the pressure on health care systems in most OECD countries. Physical activity is known to be an essential factor in improving or maintaining good health. Walking is especially recommended, as it is an activity that can easily be performed by most people without constraints. Pedometers have been extensively used as an incentive to motivate people to become more active. However, a recognized problem with these devices is their diminishing accuracy associated with decreased walking speed. The arrival on the consumer market of new devices, worn indifferently either at the waist, wrist, or as a necklace, gives rise to new questions regarding their accuracy at these different positions.

**Objective:**

Our objective was to assess the performance of 4 pedometers (iHealth activity monitor, Withings Pulse O2, Misfit Shine, and Garmin vívofit) and compare their accuracy according to their position worn, and at various walking speeds.

**Methods:**

We conducted this study in a controlled environment with 21 healthy adults required to walk 100 m at 3 different paces (0.4 m/s, 0.6 m/s, and 0.8 m/s) regulated by means of a string attached between their legs at the level of their ankles and a metronome ticking the cadence. To obtain baseline values, we asked the participants to walk 200 m at their own pace.

**Results:**

A decrease of accuracy was positively correlated with reduced speed for all pedometers (12% mean error at self-selected pace, 27% mean error at 0.8 m/s, 52% mean error at 0.6 m/s, and 76% mean error at 0.4 m/s). Although the position of the pedometer on the person did not significantly influence its accuracy, some interesting tendencies can be highlighted in 2 settings: (1) positioning the pedometer at the waist at a speed greater than 0.8 m/s or as a necklace at preferred speed tended to produce lower mean errors than at the wrist position; and (2) at a slow speed (0.4 m/s), pedometers worn at the wrist tended to produce a lower mean error than in the other positions.

**Conclusions:**

At all positions, all tested pedometers generated significant errors at slow speeds and therefore cannot be used reliably to evaluate the amount of physical activity for people walking slower than 0.6 m/s (2.16 km/h, or 1.24 mph). At slow speeds, the better accuracy observed with pedometers worn at the wrist could constitute a valuable line of inquiry for the future development of devices adapted to elderly people.

## Introduction

Physical activity is universally recognized as playing an essential role in primary, secondary, and tertiary health prevention. This has been highlighted for patients with cardiovascular disease, diabetes, or osteoporosis, among other health hazards [1]. With the widespread increase in life expectancy, these diseases become more frequent [2], thereby exerting greater pressure and generating increasing costs on the health care system. One recognized way to reduce the cost associated with this progressively frail population is to improve their independence and health by keeping them physically active [3]. Physical activity decreases the incidence of chronic diseases such as diabetes, hypertension, and obesity, among others [4], and reduces hospitalization as well as the mortality rate [5,6]. It has been demonstrated that elderly people who remain physically active reduce their risk of falling, have decreased disability, succumb less to diseases, and maintain their independence longer [7,8]. Walking is a suitable physical activity for frail individuals, as well as being one of the preferred activities among older adults [9]. Therefore, any intervention able to encourage walking activities should be promoted among this population. Goal-setting theory teaches us that measuring one’s activity, setting suitable goals, and receiving positive feedback on it is a motivating factor toward undertaking more physical activities [10]. The low cost, small size, and simple ergonomics of pedometers make them particularly suited to motivate people to stay active by monitoring their activities [11]. However, in order to successfully apply goal-setting theory, it is reasonable to expect a minimum level of accuracy from the selected pedometer. Indeed, irrelevant feedback can frustrate users and lead them to give up their objectives.

Frail individuals, such as diabetic, obese patients or those with heart failure, often walk at a slow pace (around 0.6 m/s [12] and as low as 25 m/min, or 0.4 m/s, for community ambulation [13]). At such a pace, many pedometers show a lack of accuracy with relative errors going from 30% to 60% [12,14-17]. A study on a group of patients with chronic heart failure testing the accuracy of the Omron HJ-720ITC pedometer reported an error close to 24% at 0.66 m/s, approximately 9% at 0.83 m/s, 5% at 1 m/s, approximately 3% at 1.16 m/s, and 1% at 1.33 m/s [12]. The study of Marschollek et al [14] compared 4 freely accessible pedometer algorithms on healthy people and on mobility-impaired geriatric inpatients in free walking. With healthy people, an error between 8.4% and 30.8% was observed, whereas with the geriatric population the error was between 28.1% and 62.1%. Another study [15] comparing 5 pedometers (Omron HJ-105, Yamax Digiwalker 200, SportLine330, New-Lifestyles 2000, and ActiCal) on older adults reported a mean error of 9% for all devices at a self-selected speed. This error rose to 19% at 80 steps/min, 40% at 66 steps/min, and 56% at 50 steps/min. Fitbit Ultra worn on the wrist and on the hip was tested with the Samsung GT-19300 mobile phone in a study conducted by Lauritzen et al [16]. This device was tested on 3 distinct populations: healthy adults, elderly people with normal mobility, and elderly people with reduced mobility using a rollator. The count produced for elderly people using a rollator had a greater than 60% error. Other studies have been conducted to investigate the influence of the position of the pedometer on its accuracy [18,19]. For instance, a study by Abel et al [18] tested a pedometer at 3 positions at the waist (anterior, midaxillary, and posterior) for 3 different speeds (59, 72, and 86 m/s and at own pace) and took into account the influence of the waist circumference. Whereas the placement had no influence for a low waist circumference, the posterior position was best with a high waist circumference. Another study evaluated the Yamax SW-200 pedometer in 5 different positions at the waist (left midaxillary, left midthigh, umbilical, right midthigh, and right midaxillary). The tests of using the pedometer while walking on a treadmill on flat ground, as well as ascending and descending stairs, indicated a better performance when the pedometer was positioned in the left midaxillary position [19].

Although, until recently, most pedometers were worn at the waist, a new generation entering the market offers more versatility and can be worn not only at the waist but also at the wrist or as a necklace. These new pedometer positions raise questions regarding their accuracy compared with the one worn at the waist. In order to investigate the influence of the position (wrist, waist, or necklace) in relation to the speed of movement, we conducted a comparative study of several pedometers by exploring the accuracy of their readings depending on the position of the device and the speed of movement.

## Methods

We tested 4 commercially available pedometers at 4 different walking speeds: 3 at controlled speed (0.4 m/s, 0.6 m/s, and 0.8 m/s) and 1 at uncontrolled speed (natural speed of the participants) on a normalized 100 m long floor with equidistant marks. Each experiment was videotaped at normalized speed and synchronized to the participants. Pedometers were reset between each experiment and used with full charge power. The number of steps indicated by the pedometer was compared with the number of steps manually counted using the video.

### Participants

From previous similar studies, we have identified that a minimum of 20 participants [20] is necessary to demonstrate significant differences between the experimental settings. Since slow walks simulated by adults do not produce acceleration patterns significantly different from those of frail individuals with reduced walking speed [21,22], we decided to recruit healthy people and ask them to walk at controlled paces. Participants were recruited on a voluntary basis with the only inclusion criterion being that they should be able to walk at least 500 m and not have any walking disabilities.

### Instruments

We used 4 different devices during this study: iHealth activity monitor (IH; iHealth Labs Inc, Mountain View, CA, USA), Withings Pulse O2 (WI; Withings, Issy-les-Moulineaux, France), Misfit Shine (MF; Misfit, Inc, Burlingame, CA, USA), and Garmin vívofit (GA; Garmin Ltd, Southampton, UK). Table 1 lists their specifications. We selected these devices according to the following criteria: (1) 2 devices that can be worn at several positions, can count steps during an entire day, and can be integrated into a complete solution of health monitoring, (2) 1 device that is especially small, and (3) 1 device that integrates into a wide-ranging sport ecosystem.

#### iHealth Activity Monitor

iHealth is a brand specialized in health devices such as a glucometer and a blood pressure monitor. The IH can register the total number of steps during a day, distance travelled, and calories burned. It tracks sleep quality and can be placed either at the wrist or at the waist on a belt.

#### Withings Pulse O2

Withings commercializes devices such as a blood pressure monitor, a sleep monitor, a scale, and a pedometer. The WI can be placed at the wrist, on a belt, or on a shirt collar. This device tracks the number of steps, elevation, running time, calories burned, and distance travelled. It tracks users’ sleep quality, heart rate, and blood oxygen level.

#### Misfit Shine

Misfit doesn’t offer the same range of monitoring device as the 2 previous brands. We chose the MF for its very small size, an interesting feature that favors its acceptance by elderly people who are especially sensitive to stigmatization. It can be worn at the wrist, on a belt, or as a necklace. The device tracks the number of steps, distance travelled, calories burned, and the sleep pattern.

#### Garmin Vívofit

Garmin is a brand that covers a very large ecosystem of devices for sporting activities. The GA tracks the number of steps, calories burned, distance travelled, and sleep pattern. It can only be placed at the wrist.

### Procedure

The study took place in a flat area where ground markings indicated distances. We performed the study in 2 phases. In the first phase, we requested participants to walk 200 m at their preferred pace in order to assess the performance of the pedometers at natural speed. In the second phase, participants walked for a distance of 100 m at a controlled speed wearing all pedometers simultaneously. We selected 3 different walking speeds (0.4 m/s, 0.6 m/s, and 0.8 m/s) for our experiment. The slowest speed was set to 0.4 m/s, since several studies have recognized this speed as the minimum necessary for performing everyday activities [13,23,24]. The fastest speed was limited to 0.9 m/s, since this is the limit that defines normal speed [24]. We relied on the methodology defined by Martin et al [15] and used a metronome to constrain the cadence of the walker. In order to minimize intra- and interparticipant variation, their step length was also constrained using a string attached between their legs at the level of their ankles. As footstep length and cadence are related [25], the string also enables footstep length to be limited in order to keep a natural ratio with cadence that should be adopted at a specific speed.

**Figure 1:**
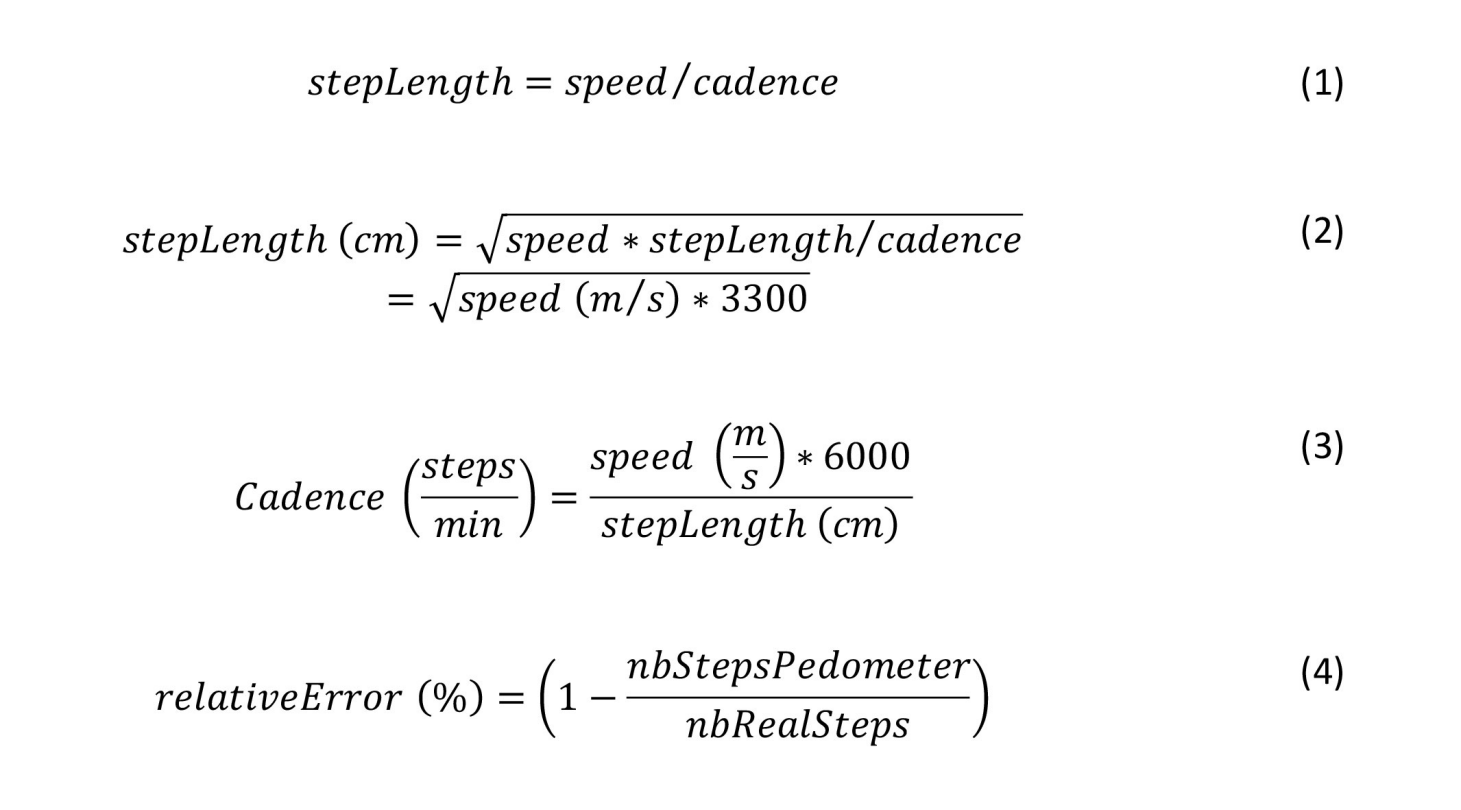
Equations for determining footstep length (stepLength; equation 1), and the relationship between speed, cadence, and footstep length (equations 2 and 3). The relative error between the real number of steps (nbRealSteps) and the number of steps registered by the pedometer (nbStepsPedometer) is calculated by equation 4.

According to research, the relation between footstep length and cadence is 0.55 steps/min [25]. Consequently, for each targeted speed, footstep length can be determined using the ratio in equation 1 expressing the relation between footstep length, speed, and cadence (Figure 1, equation 2). Once footstep length is calculated, the cadence can be simply derived by transforming the equation 1 (Figure 1, equation 3).

Based on equations 2 and 3, we calculated the various settings of the experiment, presented in Table 2.

**Table 1:** Device specifications.

Specification	Device			
	iHealth activity monitor	Withings Pulse O2	Misfit Shine	Garmin vívofit
Screen	Yes	Yes	No	Yes
Time	Yes	Yes	Yes	Yes
Steps	Yes	Yes	Yes	Yes
Calories	Yes	Yes	Yes	Yes
Distance	Yes	Yes	Yes	Yes
Sleep	Yes	Yes	Yes	Yes
Other	None	Elevation Heart rate Blood oxygen	Cycling Running Swimming	None
Position	Wrist, belt	Wrist, belt, shirt collar	Wrist, belt, necklace	Wrist
Battery life	7 days	2 days	3 months	1 year

**Table 2:** Calculated relations between speed, footstep length, and cadence.

	Speed (m/s)		
	0.4	0.6	0.8
Footstep length (cm)	36	44	51
Cadence (steps/min)	66	82	93

Each participant was encouraged to practice walking under these conditions as long as required until they considered they could walk the 100 m comfortably at the desired speed. Each walk was videotaped in order to count the number of steps precisely during the analysis stage.

### Statistical Analysis

The actual footsteps were counted manually using the videotape by 2 independent (CW and FE) observers. If the number of steps counted did not match, the counting was restarted until they corresponded. This measure was then considered as the real step count to be compared with the count returned by the pedometers.

We calculated the relative error between real number of steps and steps registered by the pedometers according to equation 4.

For each speed and each position, we calculated the mean of the error. A 1-way analysis of variance (ANOVA) was conducted to evaluate whether there was a significant difference between the position for each speed and speed for each position of the group. The confidence interval was set at 95%.

Because we collected no personal data, we did not request institutional review board approval.

## Results

A total of 21 people participated in the study, 12 women and 9 men. The average age of the participants was 34.5 years (SD 15.7).

**Figure 2:**
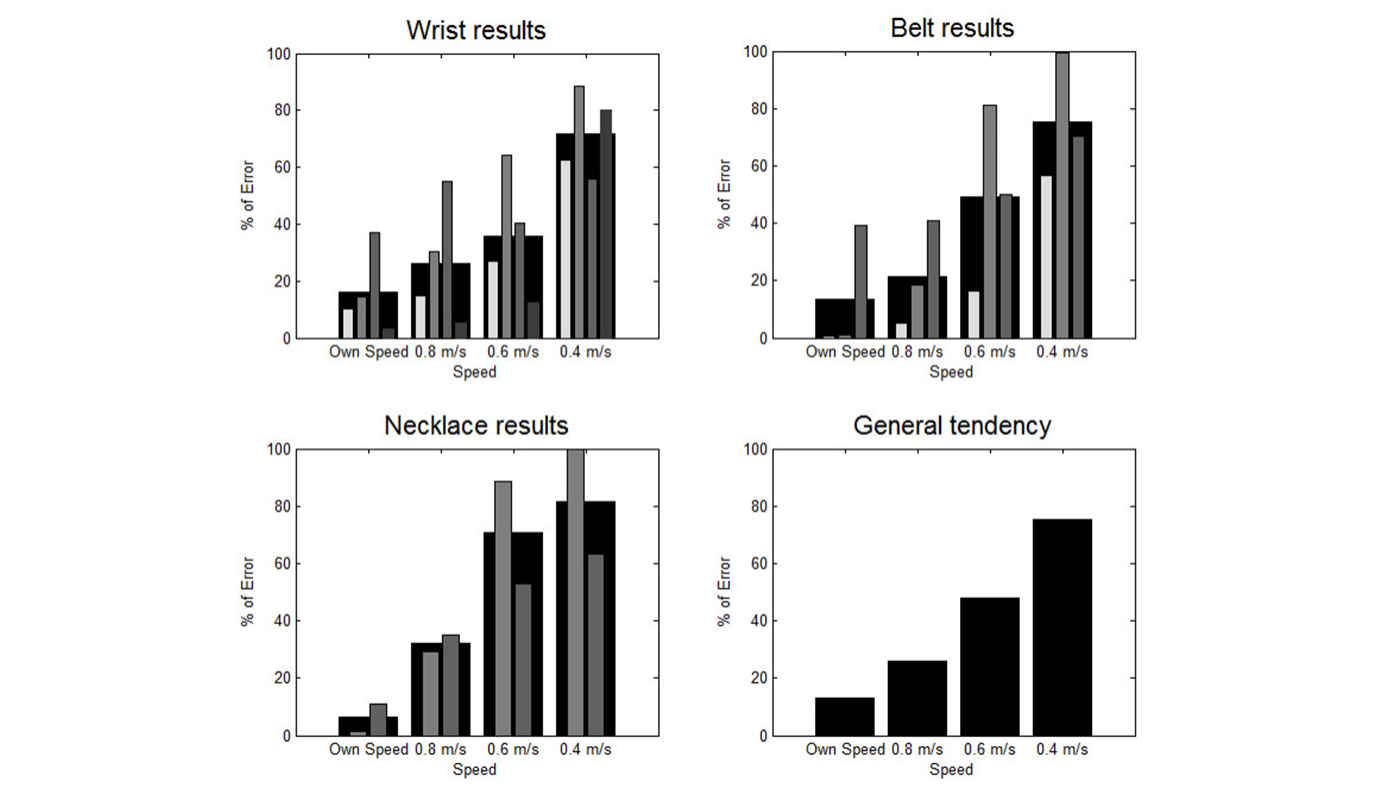
Mean relative error in terms of speed for each position (each pedometer is represented by a different shade, from the brightest to the darkest: iHealth activity monitor, Withings Pulse O2, Misfit Shine, Garmin vivofit). The largest black bar represents the average for all pedometers.

**Figure 3:**
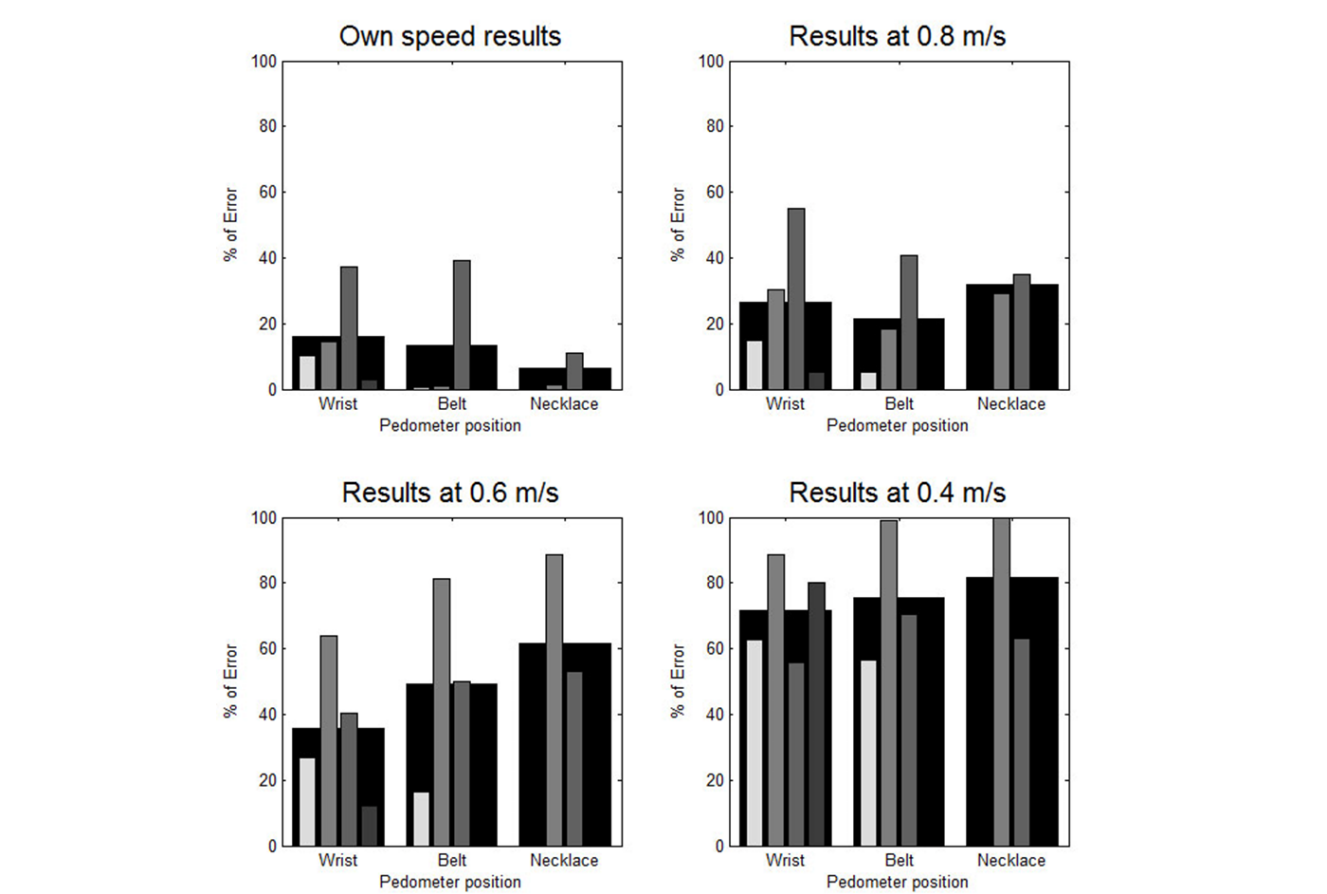
Mean relative error in terms of position for each speed (each pedometer is represented by a different shade, from the brightest to the darkest: iHealth activity monitor, Withings Pulse O2, Misfit Shine, Garmin vivofit). The largest black bar represents the average for all pedometers.

The results are presented in terms of the mean relative counting error at each speed and for each pedometer, as well as the average error for all participants (Table 3). The results are also presented graphically in Figure 2 and Figure 3. Figure 2 contains a set of bar graphs highlighting the influence of speed on accuracy by position, and Figure 3 contains a set highlighting the influence of position on accuracy by speed.

**Table 3:** Absolute mean relative error between real number of steps and steps registered by each pedometer, worn in different positions, as a percentage and standard deviation.

Pedometer	Location	Speed (m/s)			
		Natural speed	0.8	0.6	0.4
IH^a^	Wrist	10.21 (19.67)	14.79 (26.03)	26.97 (34.06)	62.64 (41.94)
	Belt	0.55 (0.67)	5.12 (11.63)	16.29 (20.99)	56.45 (27.20)
WI^b^	Wrist	14.37 (23.78)	30.20 (31.43)	64.07 (41.76)	88.51 (25.35)
	Belt	0.87 (0.80)	18.27 (29.07)	80.92 (27.00)	99.34 (3.79)
	Necklace	1.52 (2.95)	29.07 (27.55)	88.46 (25.73)	99.84 (0.50)
MF^c^	Wrist	37.16 (47.81)	55.08 (8.10)	40.18 (32.67)	55.90 (33.19)
	Belt	39.05 (48.54)	40.93 (41.62)	49.87 (39.41)	70.51 (45.08)
	Necklace	10.93 (26.05)	34.93 (34.60)	53.09 (36.18)	63.32 (35.13)
GA^d^	Wrist	3.08 (5.65)	5.31 (9.38)	12.24 (16.92)	80.14 (25.41)

^a^IH: iHealth activity monitor.

^b^WI: Withings Pulse O2.

^c^MF: Misfit Shine.

^d^GA: Garmin vívofit.

The MF pedometer generated an error higher than 30% in all cases, except at a natural pace when worn as a necklace (11%). When pedometers were worn at the wrist, the error was higher than 10% independently of the walking speed, except for GA. At a natural pace, every pedometer worn at the belt generated errors below 5%, except for MF. GA placed at the wrist and IH placed at the belt still had an error below 6% at 0.8 m/s and below 20% at 0.6 m/s. IH at the wrist and WI at the belt had an error below 20% at 0.8 m/s.

Table 4 presents the mean error in terms of position and speed, with the results of a 1-way ANOVA.

### Results According to Speed

The general tendency observed in Figure 2 highlights the correlation between the decrease of speed and the increase of mean error. This tendency was verified for every pedometer at every position except for MF at the wrist. For this pedometer, there was similar relative error at 0.4 m/s and 0.8 m/s (approximately 55%), as well as at 0.6 m/s and at natural speed (approximately 40%).

### Results According to Position

At a natural pace, when pedometers were placed at the wrist, the mean relative error was higher than when they are located at the 2 other positions (Figure 3). At the belt, pedometers were less accurate than at the collar. At 0.8 m/s, the belt position generated results with the best accuracy, followed by the wrist and then the necklace. At 0.6 m/s, the wrist position generated the lowest error, followed by the belt and the necklace positions. The same tendency was observed at 0.4 m/s.

The 1-way ANOVA showed that at each position, the mean error differed at each selected speed, except at the belt, where it was at the limit of confidence. On the other hand, 1-way ANOVA didn’t reveal a significant difference in accuracy at the various positions for a given speed.

**Table:** Mean relative error between walking speed and position of the pedometers as a percentage.

Position	Speed (m/s)				Average	*P* value
	Natural speed	0.8	0.6	0.4		
Wrist	16.21	26.35	35.87	71.80	37.56	<.05
Belt	13.49	21.44	49.03	75.43	39.85	.05
Necklace	6.23	32.00	70.78	81.58	47.65	<.05
Average	11.98	26.60	51.89	76.27		
*P* value	.49	.79	>.99	.59		

## Discussion

The influence of speed on accuracy can be clearly observed in Figure 2, regardless of which pedometer or position was selected. The mean relative error significantly increased when speed decreased until it attained more than 50% at the slowest pace. The MF placed at the wrist did not show the same tendency, since the error at 0.6 m/s was lower than the error at 0.8 m/s. We attribute this phenomenon to the strong unreliability of this pedometer, which produced a high rate of error when worn at the wrist even at a user-preferred speed.

The reason for the important error at low speed may stem from the type of algorithm used in most pedometers. In order to count steps, many algorithms rely on vertical acceleration. As the vertical acceleration diminishes according to the walking speed, it is more difficult to detect every footstep at a slow pace. MF may not use this kind of algorithm, since it did not show the same result.

We did not observe a significant influence of the pedometer position on the accuracy of the readings. Generally speaking, at normal speed, wearing a pedometer at the wrist decreased the accuracy more noticeably than wearing it at the belt or as a necklace. When the speed decreased, however, pedometers worn at the wrist had the best accuracy, and those worn as a necklace had the worst. This can be explained by the fact that, during slow walking, the vertical acceleration of the body is low but the arms are usually still moving.

It should be noted that this study was conducted on healthy adults and not on individuals walking slowly due to some impairment. Controlling the walking speed by constraining cadence and pace length using a string and a metronome can potentially change the natural way of walking. In fact, it is difficult to normalize walking because everybody reacts differently to the string between their feet. Some participants easily adopted the required cadence, whereas others needed more concentration. Nevertheless, participants were allowed to practice walking with the string using the metronome cadence until they felt comfortable and were able to adopt a natural walk before the beginning of the experiment.

It remains questionable whether the tested pedometers are suitable for a slow-walking population. Responding to this question would require identifying which level of error remains acceptable while monitoring walking activity.

Other studies have shown a similar evolution of error in terms of speed [12,15,26]), that is, the error increases when speed decreases. The Omron HJ-720ITC pedometer was tested on patients with chronic heart failure [12], producing an error close to 24% at 0.66 m/s, approximately 9% at 0.83 m/s, 5% at 1 m/s, approximately 3% at 1.16 m/s, and 1% at 1.33 m/s. Thus, when the speed increased, the error decreased. A study on older adults, comparing 5 pedometers [15], reported mean errors from 9% for all devices at a self-selected speed to 56% at 50 steps/min. At 80 steps/min, the error was 19%, and at 66 steps/min, the error was approximately 40%. A study comparing 7 pedometers [26], the DynaPort Movemonitor, Jawbone UP, Fitbit One, activPAL, Tractivity, Nike+ FuelBand, and Sensewear Armband, reported that the error increased during slow walking (around 1.6 km/h, or 0.4 m/s). But the error differences between speeds was pedometer dependent. Jawbone UP, Tractivity, Nike+ FuelBand, and Sensewear Armband showed a significant difference between slow speed and self-selected, fast speed. DynaPort Movemonitor, Fitbit One, and activPAL showed an error close to the other speeds with an error under 3.2% at every speed.

The main achievement of this study was to compare the influence of walking speed and pedometer position on the accuracy of pedometer readings. To our knowledge, ours is the first study that formally investigated this relation. This study showed that a reduction of walking speed negatively influenced the accuracy of the tested pedometers. Although this result would require a larger study to be confirmed, we observed that the position ensuring the best pedometer accuracy depended on the speed. At a normal pace, pedometers worn at the belt or as a necklace are more accurate, whereas for slow walkers, wearing pedometers at the wrist is the best choice. This study could open a valuable line of inquiry for the development of future devices for frail people, relying on the acceleration of arm movement to improve accuracy. Apart from this suggestion, this study underlines the conclusion that, before being used, a pedometer should first be assessed individually according to expected speed of movement before deciding on where to position of the device.

## Abbreviations

ANOVA: analysis of variance
IH: iHealth activity monitor
GA: Garmin vívofit
MF: Misfit Shine
WI: Withings Pulse O2
